# Effect of vitamin D on myocardial remodeling and inflammatory status in children with congenital heart disease

**DOI:** 10.55730/1300-0144.5537

**Published:** 2022-09-12

**Authors:** Sabire GÖKALP, F. Sedef TUNAOĞLU, Fatma CANBEYLİ, Semiha TERLEMEZ, Vildan ATASAYAN, Arzu ARAL, Serdar KULA, A. Deniz OĞUZ

**Affiliations:** 1 Department of Pediatric Metabolism, Faculty of Medicine, Gazi University, Ankara, Turkey; 2 Department of Pediatric Cardiology, Faculty of Medicine, Gazi University, Ankara, Turkey; 3 Department of Immunology, Faculty of Medicine, Gazi University, Ankara, Turkey

**Keywords:** Congenital heart disease, vitamin D, antiinflammatory effect

## Abstract

**Background/aim:**

Vitamin D insufficiency is a common public health problem that is often unrecognized in children with congenital heart disease, and is not generally evaluated at congenital heart disease (CHD) follow-up. Recent studies have suggested that inadequate vitamin D status may have an adverse effect on cardiovascular health. This study investigates the relationship between vitamin D levels and hemodynamic parameters in children with CHD.

**Methods:**

Included in the study 40 patients (25 females, 15 males) with CHD, who were evaluated for Ross heart failure score, vitamin D, parathyroid hormone (PTH), calcium, phosphorus, alkaline phosphatase (ALP), whole blood count (WBC) and echocardiographic measurements, and all measurements were repeated in the third month of the therapy.

**Results:**

The mean vitamin D level was 16.4 ± 6.6 ug/L before and 27.5 ± 9.9 μg/L in the third month of therapy, while the mean PTH level was 53.3 ± 34.9 pg/mL before and 43.8 ± 21.4 pg/mL in the third month of therapy. The mean WBC was 8084 ± 2324/μL before and 7378±1893/μL in the third month of the therapy, and the mean platelet (PLT) count was 280,897 ± 80,119/μL before and 307,179 ± 60,202/μL in the third month of the therapy. The mean ejection fraction (EF) was 64% ± 7.2% before and 66.7% ± 6.2% in the third month of therapy, while the right ventricle (RV) myocardial performance index (MPI) was 32.1% ± 6.7% before and 28.9% ± 6.5% in the third month of the therapy. IL10 level was increased in four patients in the third month of therapy. A statistically significant decrease in PTH level and WBC, and an increase in PLT number and IL-10 level were detected by the therapy. Furthermore, echocardiographic findings revealed a statistically significant increase in EF and a decrease in RVMPI attributable to the therapy.

**Conclusion:**

The decreased levels of PTH, which is a proinflammatory marker, the increases in IL-10, which is an antiinflammatory cytokine, and the decreases in the number of WBC resulting from vitamin D treatment demonstrate the antiinflammatory effects of vitamin D. An improvement in EF means improvement in left ventricular contractility, while a decrease in RV MPI has been shown to improve the systolic and diastolic function of the right ventricle. These results suggest that vitamin D therapy has a positive effect on the heart, and so vitamin D levels should be evaluated during the routine follow-up of congenital heart disease.

## 1. Introduction

Vitamin D is one of the oldest known hormones, showing its effects through its receptors. Vitamin D receptors (VDR) are found in immune system cells, epidermal keratinocytes, vascular smooth muscle, and cardiac myocytes, and play a regulatory role in cell proliferation and differentiation [1]. Vitamin D reduces the synthesis of proinflammatory cytokines while improving the synthesis of antiinflammatory cytokines, and increased levels of proinflammatory cytokines in children with congenital heart disease contribute to the development of cardiac inflammation and heart failure. Interleukin-10 (IL-10) is an antiinflammatory cytokine that suppresses the proinflammatory cytokines, and has a negative effect on heart contraction at low levels [2].

In the presence of vitamin D deficiency, PTH serum levels become elevated – a condition that is associated with cardiac hypertrophy and fibrosis. Increased proinflammatory cytokine and PTH levels have an adverse effect on cardiac function and the prognosis of CHD [1,3,4]. This study investigates the role of vitamin D in the follow-up of CHD, as a factor that is not routinely evaluated in such cases.

## 2. Materials and method

Included in the study were 40 patients with congenital heart disease who were admitted to the outpatient clinic of the Pediatric Cardiology Department of Gazi University. This study was conducted according to the principles of the 1975 Declaration of Helsinki, and ethics approval was obtained from the Gazi University Faculty of Medicine ethics committee before starting the study. Informed consent was taken from the parents of all those involved in the study. The patients were questioned about the therapies they were taking, and their dietary patterns, and vitamin D prophylaxis. The patients’ vital signs were recorded, a general physical examination was performed by the same pediatrician and the Ross heart failure scores were recorded.

Each patient submitted a 5 mL venous blood sample, which were placed in vacuum tubes and centrifuged for 5 min at 5000 rpm. Lipemic and hemolyzed sera were not studied. On the same day, complete blood count, calcium, phosphorus, alkaline phosphatase, 25 hydroxyvitamin D and parathormone levels were measured.

ELISA kits (IL-10 ELISA, Lot No. 74743547A, Cat No. KHC0101; Invitrogen, ThermoFisher Scientific, USA) were used for the IL-10 measurements of the blood samples, which were stored at –80 °C.

Echocardiographic studies were performed by the same pediatric cardiologist. All measurements were made in accordance with American Echocardiography Society recommendations using a Vivid E9 ECO device obtained from General Electric Medical Systems (USA), fitted with a 3.5–5 MHz probe [5]. For the measurements, images were taken from the subcostal, parasternal long axis, short axis, apical four chambers, five chambers, and suprasternal positions; and M-mode, 2-dimensional, color Doppler and tissue Doppler echocardiographic examinations and systolic and diastolic functions, as well as right and left ventricular tissue myocardial performance indexes (MPI), were measured. Left ventricular mass and left ventricular mass indexes were calculated and Z scores were determined.

Left ventricular mass (LVM) is calculated using the Devereux and Reishek formula:


$$\begin{array}{l} \text{Left ventricular mass }(\text{g})=1.04\times [({\text{LVED}}^{★}+{\text{IVST}}^{★}+{\text{RWT}}^{★})]\;3-13.6\\ \text{Left ventricular mass index }(\text{LWMI})=[(\text{LV mass}/\text{body surface area}) \end{array}$$

(*LVED: Left ventricular end-diastolic diameter, IVST: Interventricular septum thickness, RWT: Relative wall thickness)

Serum concentrations of 25 hydroxyvitamin D below 20 ug/L were defined as vitamin D deficiency; and serum concentrations of 25 hydroxyvitamin D between 20–30 ug/L were defined as vitamin D insufficiency. Patients with vitamin D deficiencies/insufficiencies were started on a vitamin D treatment plan, as follows:

- 2000 IU/day for children with 25 hydroxyvitamin D levels below 20 ug/L or 50,000 IU once a week for 6 weeks, after which therapy was continued at 400–1000 IU/per day.
- Vitamin D was given at 800–1000 IU/day in individuals with 25 hydroxyvitamin D levels between 20 ng/mL and 30 ng/mL for 12 weeks [6].

Following the completion of the treatment, the Ross scores, echocardiographic examination results, serum IL-10, vitamin D, parathyroid hormone, calcium, phosphorus and alkaline phosphatase levels were reexamined.

The research data were evaluated using IBM SPSS Statistics for Windows (Version 22.0. Armonk, NY: IBM Corp.). Descriptive statistics were presented as mean ± standard deviation or minimum-maximum, frequency distribution, and percentage. The normal distribution suitability of the variables was examined using visual (histogram and probability plots) and analytical methods (Shapiro-Wilk Test). A Wilcoxon signed rank test was used for statistical analyses. The statistical significance level was accepted as p < 0.05.

## 3. Results

The mean age of the patients was 109.8 ± 47.4 months and 62.5% of patients were female. The average height of the patients was 133.9 ± 23.3 cm, the mean body weight was 34.0 ± 17.2 kg and the mean BMI was 17.7 ± 4.0 kg/m^2^. The mean systolic blood pressure was 98 ± 13.4 mmHg and the mean diastolic blood pressure was 62.8 ± 12.1 mmHg (Table 1). All patients were assessed as Class I (asymptomatic) according to the Ross classification for the clinical evaluation of heart failure.

The most frequent diagnoses were ASD 37.5% (n = 15), VSD 22.5% (n = 9), and tetralogy of Fallot 10% (n = 4) (Table 2).

The duration of treatment was 12 weeks. All patients completed the therapy and were reexamined after 12 weeks, when vitamin D levels and thrombocyte numbers were found to be significantly increased, while the PTH value and WBC had decreased significantly from the values measured before treatment (p < 0.001, p = 0.048, p = 0.023, p = 0.017, respectively). There was no statistically significant difference between the calcium, phosphorus, ALP and hemoglobin values before and after treatment (Table 3). The IL-10 value of all the patients before treatment was 0 (zero), while after treatment the average IL-10 values of the four patients were increased (mean 14.6 ± 10.0 pg/mL).

EF, RVS’, and RVA’ values increased significantly while RVMPI decreased significantly after the therapy (Table 4).

## 4. Discussion

There have been many studies of patients with heart failure associated with congenital heart disease and vitamin D deficiency. Shedeed et al., evaluated patients with congestive heart failure and vitamin D deficiency in two groups, which were compared after at the 12 weeks of therapy. The placebo-treated group was compared with the vitamin-D-treated group, and a significant decrease was noted in the parathormone level with the increase in vitamin D levels, as Shedeed’s study concurred with the findings of the present study [2]. In another study, including adult patients with pulmonary hypertension, pulmonary artery pressure was significantly higher in the group with vitamin D deficiency. Parathormon levels have also been reported to be higher in the group with vitamin D deficiency. It has been suggested that high PTH levels are associated with an increase in pulmonary artery pressure [3]. It is known that increased parathormone levels are associated with vascular proliferation, cardiac hypertrophy, and fibrosis, and it has been suggested that the decrease in parathormone levels associated with vitamin D treatment is indicative of the cardioprotective effect of vitamin D [7].

Studies have shown that the majority of tissue contains vitamin D receptors, and that changes in vitamin D levels can affect tissue function. There have been a number of studies claiming the existence of a relationship between vitamin D and hemogram parameters, and other studies indicating that there is no relationship. Vitamin D affects tissue through VDR, which is encoded by chromosome 12 and varies widely between individuals as a result of polymorphism. VDR is present in the hematopoietic system, monocytes, active T and B lymphocytes, thymocytes, and different precursor cells, aside from the classical intestinal, renal and bone tissues [8]. It is thought that vitamin D may act on very different tissues in the body, including the hematopoietic system. In the present study, WBC levels were seen to decrease and PLT levels to increase after the therapy. The results of studies investigating the effect of vitamin deficiency on hematological parameters are contradictory. In a study by Kebabçılar et al., a negative correlation was identified between vitamin D and MPV, D-dimer and aPTT levels, but no relationship between vitamin D and WBC [9]. In the study by Yıldırım et al., there was no correlation with vitamin D level and albumin, C-Reactive Protein (CRP), erythrocyte sedimentation rate (ESR) and WBC [10]. In the study by Öztürk et al., no correlation was identified between vitamin D levels and hemogram values ​​[11]. In our study the number of white cells decreased significantly with treatment, which thought the efficacy of vitamin D as an antiinflammatory immunomodulator. There have been studies suggesting that this activity is not associated only with 25-hydroxyvitamin D levels, and that other unknown and more complex processes may be involved. In a study of mice, low 1.25 (OH) _2_D3 has been reported to increase TGF-β1 and IL-4 production [12].

Bockow et al. reported an increase in platelet counts of vitamin D given with steroids and hydroxychloroquine in patients with immuno-thrombocytopenic purpura (ITP) were observed. The number of platelets decreased with the removal of vitamin D, and a further increase was observed with the addition of vitamin D treatment. The authors attributed this increase in platelet count to the immunomodulatory feature of vitamin D, and suggested also that the decrease in CD4 + cell proliferation and the increase in T regulatory cells resulting from Vitamin D therapy caused a decrease in the amount of autoantibodies, leading to an increase in the number of platelets [12,13]. In the present study, we thought that an increased platelet count was detected following vitamin D therapy as a result of the immunmodulatory effect of vitamin D.

In our study, when IL-10 levels from before and after treatment were compared, a significant increase was noted in IL-10 levels in four patients at the end of treatment. This increase in IL-10–an antiinflammatory cytokine- levels after the treatment confirms the antiinflammatory activity of vitamin D. In a study by Schleithoff et al., adult patients being followed for heart failure and taking vitamin D were found to experience a significant increase in IL-10 levels, a decrease in PTH and TNF-α levels, and an increase in 25-hydroxyvitamin levels at the end of 6 weeks [14].

There have been many adult studies reporting a relationship between heart failure and vitamin D deficiency. Dalbeni et al. separated two groups of adult patients being followed for heart failure, and when the two groups were compared, a significant increase in EF was noted in the group being given vitamin D, leading the authors to suggest that vitamin D had a positive effect on the heart [15].

The myocardial performance index (MPI) is a parameter that allows a global evaluation of ventricular systolic and diastolic function. It has been suggested that the decrease in MPI noted after vitamin D treatment may be attributed to the stronger contraction of the right ventricle by vitamin D therapy in our study.

Inflammatory mediators and PTH are known to play a critical role in the ventricular “remodeling” process and in the prognosis of heart failure. Vitamin D has been shown to reduce PTH and inflammatory mediators, which may be protective for remodeling in patients with heart failure [16].

## 5. Conclusion

In the present study, a decrease in the number of white blood cells and PTH levels, an increase in the IL-10 level and left ventricular EF, and a decrease in the right ventricular MPI value were achieved with vitamin D therapy. These results reveal a positive effect of vitamin D supplementation on the heart. Given the low number of studies reporting positive results on the heart through vitamin D therapy in children, the results can be considered important. Vitamin D should be included in the prophylaxis and follow-up protocols of patients with congenital heart disease, although further studies are needed to support these findings.

## Figures and Tables

**Table 1:** Characteristics of the patients.

	±S (min-max)
**Age (month)**	109.8 ± 47.4 (7–204)
**Length (cm)**	133.9 ± 23.3 (67–175)
**Weight (kg)**	34.0 ± 17.2 (7–75)
**BMI (kg/m** **^2^** ** )**	17.7 ± 4.0 (12.0–33.2)
**Systolic blood pressure** (mmHg **)**	98 ± 13.4 (70–120)
**Diastolic blood pressure** (mmHg)	62.8 ± 12.1 (35–90)

χ̄: Average; S: Standard deviation, mean ± SD (min-max)

**Table 2:** The diagnoses of the patients on vitamin D treatment.

	n (%)
ASD	15 (37.5)
VSD	9 (22.5)
Tetralogy of fallot	4 (10.0)
Pulmonary stenosis	3 (7.5)
Aortic stenosis	3 (7.5)
Coarctation of aorta	2 (5.0)
Transposition of great arteries	1 (2.5)
PDA	1 (2.5)
PFO	1 (2.5)
AVCD	1 (2.5)

**Table 3:** Laboratory values before and after treatment.

	N	Before treatment	After treatment	p*
		±S (min-max)	±S (min-max)	
**Vitamin D(ug/L)**	39	16.4 ± 6.6 (7–30)	27.5 ± 9.9 (10–62)	<0.001
**PTH (pg/ml)**	39	53.3 ± 34.9 (9.5–148)	43.8 ± 21.4 (10–112)	**0.048**
**Calcium (mg/dl)**	39	9.8 ± 0.4 (8.7–10.7)	9.6 ± 0.4 (9–10.3)	0.069
**Phosphorus (mg/dl)**	37	4.8 ± 0.6 (3.6–6.1)	4.7 ± 0.5 (4–6)	0.108
**ALP (U/L)**	39	209.2 ± 76.2 (67–442)	218.4 ± 76.5 (66–400)	0.490
**Hemoglobin (g/dL)**	39	12.2 ± 2.01 (3.8–15.8)	12.6 ± 1.3 (9.5–16)	0.093
**WBC (/** **μ** **L)**	39	8084.2 ± 2324.3 (4180–17,900)	7378.2 ± 1893.5 (4800–14,000)	**0.023**
**Thrombocyte(/** **μ** **L)**	39	280897 ± 80119.5 (143,000–485,000)	307,179 ± 60,202.2 (186,000–446,000)	**0.017**

N: Number of patients; χ̄: Average; S: Standard deviation; PTH: Parathormone; ALP: Alkaline phosphatase; WBC: White blood cell

* Wilcoxon Marked Rank Test

**Table 4:** Comparison of ECHO measurements.

	N	Before treatment	After treatment	P
		±SS (min-max)	±SS (min-max)	
**LVMI (gr/m** **^2^** **)**	38	45.6 ± 18.6 (29–143)	45.6 ± 16.2 (24.1–115)	0.988*
**Z score**	38	0.6 ± 1.3 (−2.3; 4.2)	0.7 ± 1.2 (−1.6; 4.1)	0.731*
**LVCD (mm)**	38	3.7 ± 0.6 (2.2–5.1)	3.7 ± 0.6 (2.1–5)	0.638**
**LVCS (mm)**	38	2.4 ± 0.4 (1.4–3.6)	2.5 ± 0.3 (1.7–3.2)	0.446**
**FK (%)**	38	35.7 ± 10.7 (3.3–79)	36.2 ± 4.8 (27–46)	0.059*
**EF (%)**	38	64.0 ± 7.2 (47–80)	66.7 ± 6.2 (53–78)	**0.028** **
**IVSD (mm)**	38	0.8 ± 0.1 (0.5–1.2)	0.8 ± 0.2 (0.4–1.3)	0.708**
**IVSS (mm)**	38	0.9 ± 0.2 (0.1–1.7)	0.9 ± 0.2 (0.6–1.5)	0.617*
**Ao/PA**	37	1.1 ± 0.2 (0.7–1.6)	1.1 ± 0.3 (0.7–1.8)	0.264**
**Ao/LA**	38	0.8 ± 0.2 (0.5–1.4)	0.8 ± 0.1 (0.4–1.2)	0.446**
**LVS**′ **(m/s)**	38	0.1 ± 0.1 (0.06–0.9)	0.09 ± 0.02 (0.05–0.1)	0.530*
**LVE**′ **(m/s)**	38	0.1 ± 0.0 (0.1–0.2)	0.1 ± 0.03 (0.03–0.2)	0.082*
**LVA**′ **(m/s)**	38	0.06 ± 0.02 (0.04–0.1)	0.06 ± 0.03 (0.03–0.2)	0.437*
**LV MPI (%)**	36	30.3 ± 7.7 (17–51)	29.5 ± 6.5 (16–44)	0.638**
**RVS**′ **(m/s)**	38	0.09 ± 0.02 (0.04–0.1)	0.10 ± 0.02 (0.05–0.1)	**0.037** *
**RVE**′ **(m/s)**	38	0.1 ± 0.04 (0.06–0.2)	0.1 ± 0.04 (0.09–0.2)	0.270**
**RVA**′ **(m/s)**	38	0.07 ± 0.03 (0.04–0.1)	0.08 ± 0.04 (0.04–0.3)	**0.032** *
**RV MPI (%)**	36	32.1 ± 6.7 (16–47)	28.9 ± 6.5 (19–44)	**0.035** **

N: Number of patients; χ̄: Average; S: Standard deviation;

* Wilcoxon Marked Rank Test

** Paired Sample T Test

LVMI: left ventricular mass index, LVCD: left ventricular end systolic diameter, LVCS: left ventricular end diastolic diameter, FK: fractional shortening, EF: ejection fraction, IVSD: interventricular septum diastolic thickness, IVSS: interventricular septum systolic thickness, Ao: Aortic, PA: Pulmonary artery, LV: Left ventricle, RV: Right ventricle, MPI: Myocardial performance index
